# Viral strategies for targeting spinal neuronal subtypes in adult wild-type rodents

**DOI:** 10.1038/s41598-022-12535-4

**Published:** 2022-05-23

**Authors:** Jaspreet Kaur, Rune W. Berg

**Affiliations:** Department of Neuroscience, Faculty of Health and Medical Sciences, University of Copenhagen, Blegdamsvej 3, 2200 Copenhagen, Denmark

**Keywords:** Motor control, Neural circuits, Genetics, Neuroscience

## Abstract

Targeting specific subtypes of interneurons in the spinal cord is primarily restricted to a small group of genetic model animals. Since the development of new transgenic model animals can be expensive and labor intensive, it is often difficult to generalize these findings and verify them in other model organisms, such as the rat, ferret or monkey, that may be more beneficial in certain experimental investigations. Nevertheless, endogenous enhancers and promoters delivered using an adeno-associated virus (AAV) have been successful in providing expression in specific subtypes of neurons in the forebrain of wildtype animals, and therefore may introduce a shortcut. GABAergic interneurons, for instance, have successfully been targeted using the *mDlx* promoter, which has recently been developed and is now widely used in wild type animals. Here, we test the specificity and efficiency of the mDlx enhancer for robust targeting of inhibitory interneurons in the lumbar spinal cord of wild-type rats using AAV serotype 2 (AAV2). Since this has rarely been done in the spinal cord, we also test the expression and specificity of the CamKIIa and hSynapsin promoters using serotype 9. We found that *AAV2-mDlx* does in fact target many neurons that contain an enzyme for catalyzing GABA, the GAD-65, with high specificity and a small fraction of neurons containing an isoform, GAD-67. Expression was also seen in some motor neurons although with low correlation. Viral injections using the CamKIIa enhancer via AAV9 infected in some glutamatergic neurons, but also GABAergic neurons, whereas hSynapsin via AAV9 targets almost all the neurons in the lumbar spinal cord.

Genetic tools have been instrumental for teasing apart circuits in the central nervous system^1^. Targeting specific sub-populations of neurons have helped addressing questions whether the functional organization of the nervous system can be linked to the genetic identity of neurons. Such investigations are often accomplished using transgenic animals, which are expensive to acquire, labor intensive to breed and often restricted to only a single model organism, which is most often the mouse. As a consequence, it is difficult to assess the translational potential of these findings in other model organisms that are less mainstream albeit closer to humans, such as the rat, ferret or monkey. Therefore genetic tools that can be delivered via virus to target specific cellular subtypes using an endogenous promoter or enhancer without the need of transgenic animals would not only be helpful, but also scientifically important by ensuring diversity in model organisms. Cell-type genetic drivers that are based on endogenous gene expression could also be used in combination with the transgenic tools to increase the options of manipulations, e.g. to combine optogenetics and calcium imaging, or to image two populations simultaneously.

**Figure 1:**
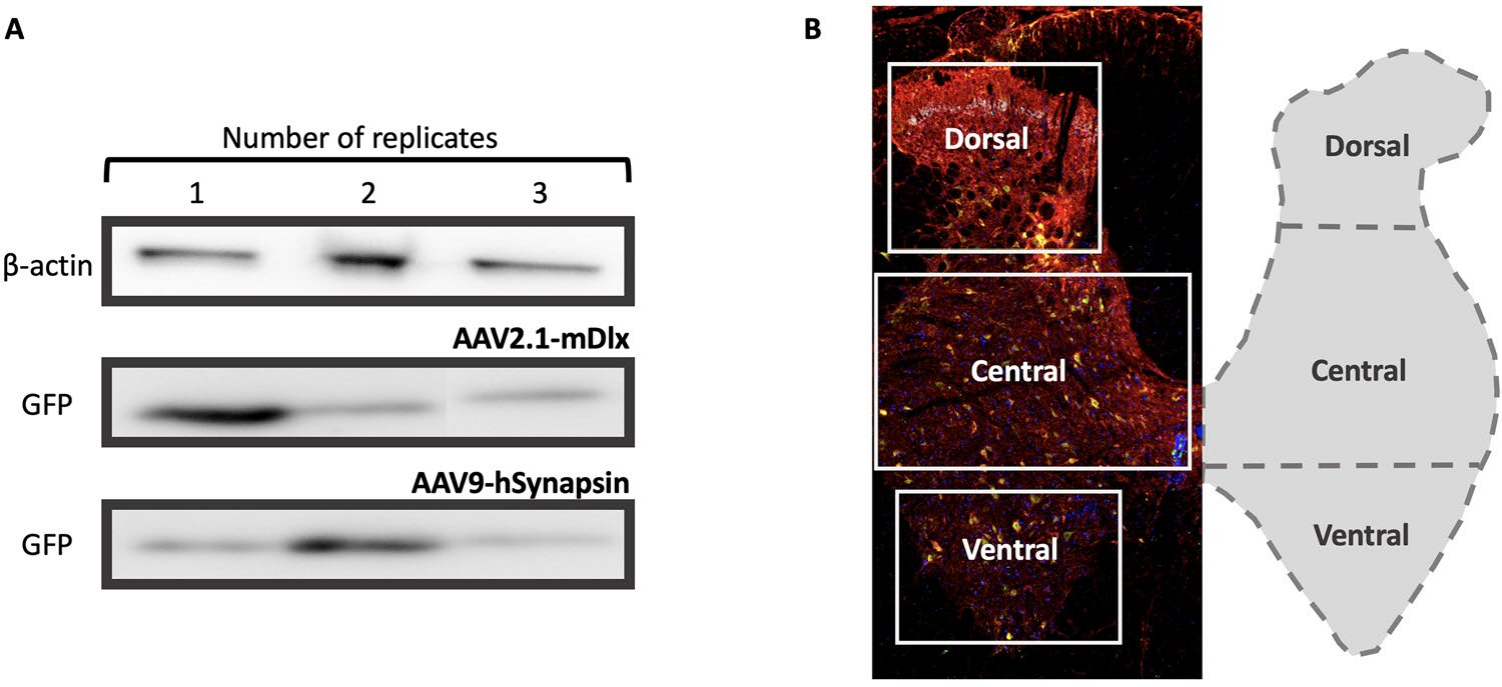
Expression of AAV viruses in the lumbar spinal cord of wild-type Wistar adult rats. (**A**) Western blots of the images confirming expression of two viruses, AAV2.1-mDlx-GCaMP6f-Fishell-2 (N = 3 rats) and AAV9-hSynapsin-soCoChR-GFP (N = 3 rats) with beta-actin as the reference protein. (**B**) Confirmation of expression was performed by visual inspection of the fluorescent reporter, where after immunohistochemistry was performed and cell colocalization and counting was performed in three dorsoventral regions of the lumbar cord (D, 560 $\times$ 720  $\upmu {\text {m}}^{2}$), central (C, 590 $\times$  1070 $\upmu \text {m}^{2}$) and ventral (V, 460 $\times$ 770 $\upmu \text {m}^2$). Red color represents GAD65, green GFP (mDlx), cyan GAD67 and blue DAPI. Full length western blots are shown in the supplementary Fig.  1A,B.

Generic endogenous promoters like Ca$^{2^+}$/calmodulin-dependent protein kinase II$\alpha$ (CamKIIa)^2,3^, human synapsin 1 gene (hSyn1)^4^ have been applied extensively for targeting mature neurons of wild-type animals. Nevertheless, the former targets primarily excitatory neurons and the later targets all neurons non-specifically^5^, and alternative promoters to target other neuronal cell types are equally in demand. Various serotypes of AAVs (1, 2, 5, 8, 9) containing the CamKII and hSynapsin1 promoters have been applied in the marmoset, mouse and macaque cortex and this indicated that CamKII was uniformly targeting excitatory neurons than hSyn1^5^. CamKIIa expresses in pyramidal and granule neurons of hippocampus, neocortex mainly in layer II, III and IV, cerebellum, thalamus, hypothalamus, basal ganglia and spinal trigeminal nucleus^6^. However, a large number of CamKIIa-expressing neurons in the olfactory bulb and some layer I cells in the neocortex have reported to be GABAergic^6^. The hSynapsin1 promoter has shown substantial expression in striatum and thalamus in the adult rat brain^4^, and in the whole brain, peripheral nerves and spinal cord of neonatal mice^7^.

The novel transcriptional enhancer mDlx is based on the *distal-less* (Dlx) homeobox gene group, which is a key regulator for inhibitory interneurons^8^. The Dlx genes are important in both invertebrates and vertebrates^9^ and are involved in expression of interneurons in the telencephalon, that primarily use Gamma-amino-byturic acid (GABA) as neurotransmitter^10^. The Dlx genes are necessary for expression of the enzyme to catalyze GABA (GAD65 and 67) and general GABAergic synaptogenesis and dendritogenesis^8,10^. The mDlx enhancer has been demonstrated useful in investigations of e.g. the ferret visual cortex^11^, but it is unknown whether the specificity is found in the spinal cord^12^. There is a large heterogeneity in spinal interneurons^13,14^, for which the functional role is unclear. Inhibitory interneurons in the spinal cord has been proposed to be providing a counter-balance of excitation^15–17^ yet their role also remain unclear. The Dlx homeobox gene group is known to mark the neural crest, the telencephalic structures and placodes and primarily expressed in telencephalic structures^18^, hence it is unsure whether this enhancer will be specific in the spinal cord.

Here, we tested the mDlx enhancer with AAV2 for marking inhibitory interneurons in the spinal cord, using an adeno-associated virus of serotype 2, which was injected in the lumbar spinal cord of wild-type rats. Furthermore, we tested the promoters hSynapsin1 and CaMKIIa using serotype 9. The latter is known to target primarily excitatory neurons in the forebrain^2,3^, but the expression characteristics in the spinal cord are less well-established. First we verified the expression of AAVs carrying these promoters and then quantify the expression in the dorso-ventral axis (Fig. 1). The expression specificity was evaluated using immunohistochemistry. It should be noted that part of the expression specificity can depend not only on the promoter/enhancer, but also on the serotype of the AAV.

## Results

An initial verification of viral expression in the spinal cord was performed using western blots, which showed substantial expression of two AAV viruses (AAV2-mDlx and AAV9-hSynapsin) used in the study (Fig. 1, Supplementary Fig.  1A,B). Further, the expression was further confirmed by sectioning the fixed spinal cords and visualizing under the fluorescent microscope before performing immunostaining. Expression of the an AAV9 virus with CamKIIa promoter was possible to confirm by visual inspection using fluorescent microscope of spinal cord sections. After the confirmation of expression immunohistochemistry was performed on the appropriate slices.

**Figure 2:**
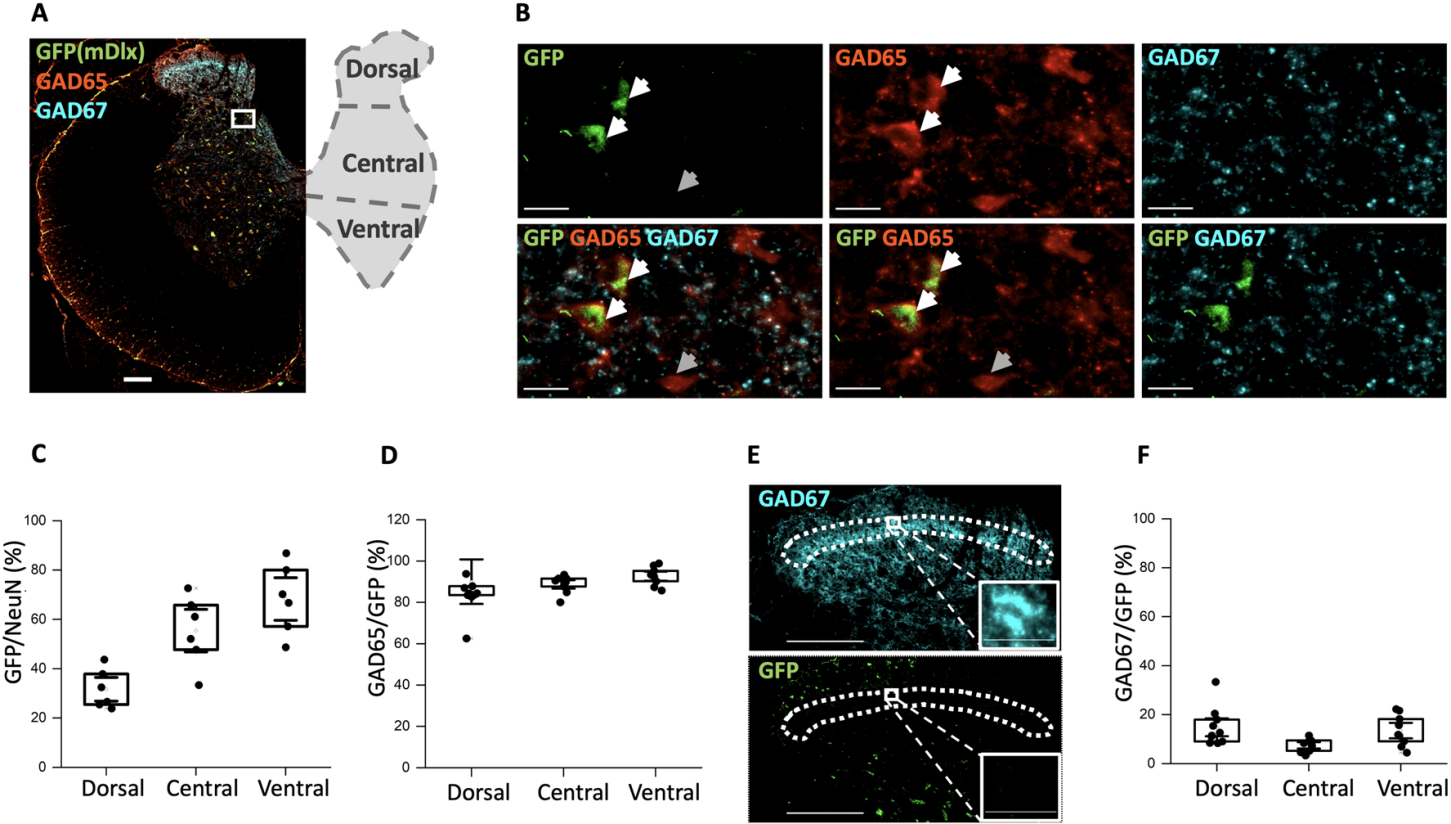
The AAV2-mDlx enhancer primarily expressed in GAD65-containing neurons in the lumbar spinal cord. (**A**) Sample tissue section and illustration of the spinal cord showing a distinct layer of GAD67-containing synaptic terminals and neurons (cyan) whereas GAD65-containing neurons are more dispersed (red). The AAV2-mDlx-driven viral expression is shown in green (GFP). (**B**) A highlighted section (panel **A**) indicates co-localization of GFP and GAD65, but not GAD67. There are also instances of GAD65+ cells where GFP was not expressed (dim arrow). (**C**,**D**) Fraction of co-localization of GFP+ cells and cells containing NeuN (**C**, N = 2 rats, n = 3 sections/rat) and GAD65 (**D**, N = 3 animals, n = 3 sections) in the three dorso-ventral regions. (**E**) The dense layer of GAD67-containing neurons in the dorsal horn (substantia gelatinosa) has little or no overlap with GFP. **(F)** Cell count (shown as %) of the co-expression in the three regions between GAD67 and GFP positive cells (N = 3 animals, n = 3 sections/animal).

**Figure 3:**
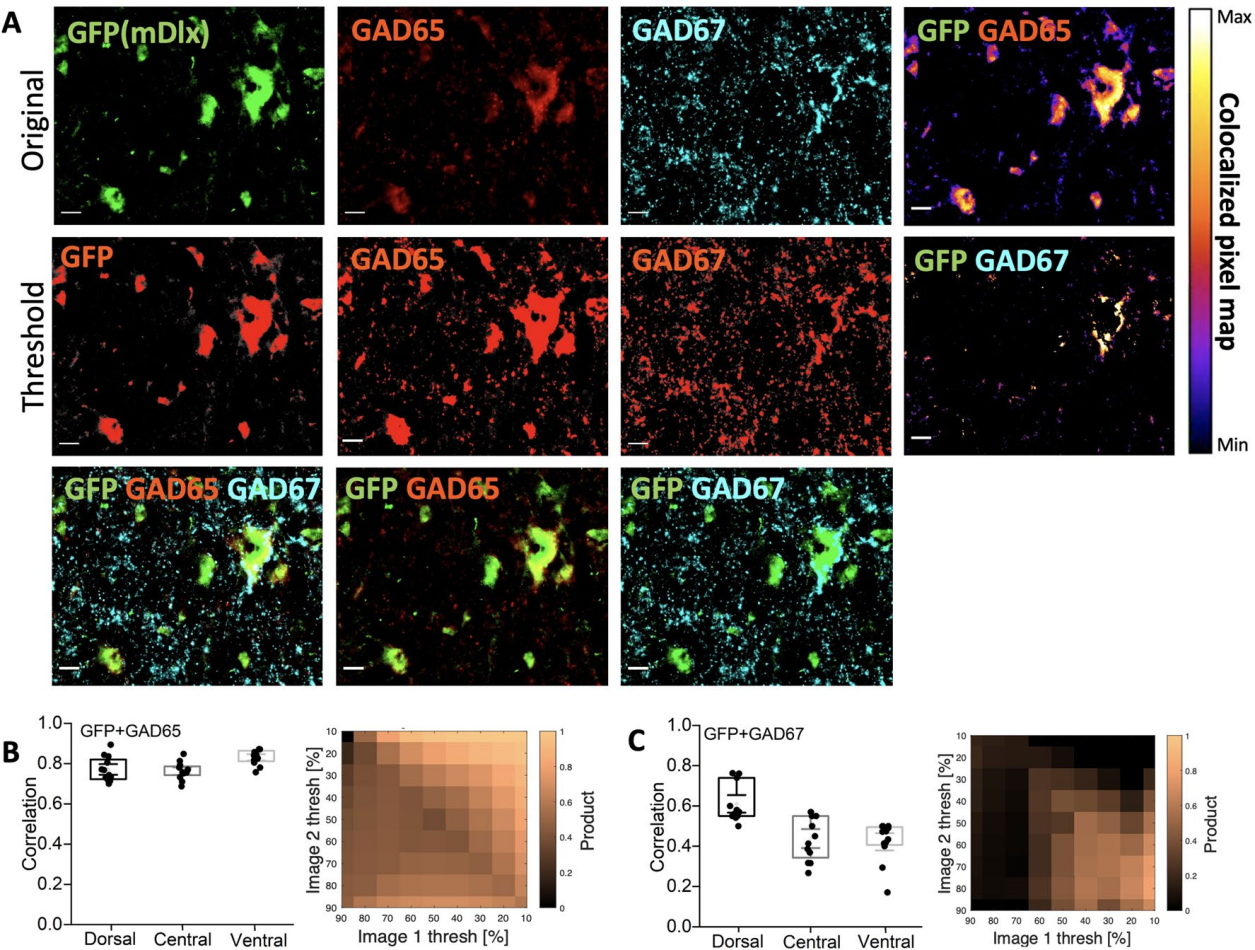
AAV2-mDlx has expression in GABAergic neurons, specifically the GAD65+ neurons. (**A**) Top row: original images of mDlx reporter (GFP, green) and GAD65 (red) and GAD67 (cyan) and a colocalization pixel map of GFP/GAD65 (right). Middle row: thresholded versions of top row, with the overlap in heat map (right) (scale bar 20 $\upmu$m). Bottom row: overlap of GFP/GAD65/GAD67, GFP/GAD65 and GFP/GAD67, respectively. (**B**,**C**) Varying the threshold and calculating the co-localization as correlation (**C**). Note N = 4 animals, n = 3 sections/animal.

### AAV2-mDlx expression primarily in GAD65 neurons

We tested whether the mDlx enhancer with AAV2 that has been developed to target GABAergic interneurons in the forebrain^12^ can also target GABAergic neurons in the lumbar spinal cord of adult rats. The virus carried a fluorescent reporter, the green fluorescent protein (GFP). First, we verified the targering and expression of GABAergic neurons in the mouse brain (Supplemental Fig.  2). For verification of GABAergic identity of infected cells in mice prefrontal cortex we used the Pax2 transcription factor, which has been associated with inhibitory interneurons^19^. Pax2 showed colocalization with GFP labelled neurons (Supplemental Fig.  2) whereas no colocalization with GAD67 biomarker was found.

Next, we tested the AAV2-mDlx in rat lumbar spinal cord and the degree of labeling of all neurons in the section. Hence, GFP (to label mDlx expressed neurons) was compared with a general neuron biomarker (NeuN, Fig. 2C). The fraction of neurons with AAV2-mDlx-driven expression compared with NeuN was found to be approximately one third in the dorsal, one half in the central and more than half (60%) in the ventral part of the spinal cord.

Next, the GFP-expressing neurons was compared with inhibitory cells carrying the isoforms of the enzyme glutamate decarboxylase 65 (GAD65) and 67 (GAD67) as GABAergic biomarkers (Fig. 2). We found that majority of GFP+ neurons co-localized with GAD65+ neurons (white arrows). There were instances of a GFP-negative cell that was stained for GAD65 (dim arrow). In total, approximately 80% of GFP+ neurons were co-localized with GAD65+ in the dorsal (D), central (C) and ventral (V) regions (Fig. 2A,B,D). In contrast, neurons expressing the isoform, GAD67+, had only 5–10% of co-localization with the GFP+ neurons (Fig. 2E,F). GAD67 were present either as punctuate structures or in the somata. Neurons and synaptic terminals with clear staining for GAD67 were abundantly present in the lamina 2 (substantia gelatinosa), and virtually none of these had expression of GFP (Fig. 2E). To further investigate the co-expression between GFP and GABAergic neurons, we analysed the data using a measure of protein colocalization where values of the original images (top row, Fig. 3A) above a certain threshold in one image was multiplied by the similar values in the other image (middle row, Fig. 3A). This provides an objective measure of the correlation of the location of two fluorescent proteins^20^, whose value depends on the choice of threshold (Fig. 3B,C). Regardless of the choice, the values were quite high for GFP/GAD65+ (Fig. 3B) and average to low for GFP/GAD67+ neurons (Fig. 3C) which are shown as co-localization pixel maps (Fig. 3A, top and middle right images). To further inspect the cell type, we used Pax2 and found that GFP+ neurons showed fluorescent correlation of 0.6 (Supplementary Fig.  3A–D) whereas cell counting analysis showed approximately 15–35% of Pax2+ neurons in the D, C and V regions co-localize with GFP+ neurons (Supplementary Fig.  3E).

**Figure 4:**
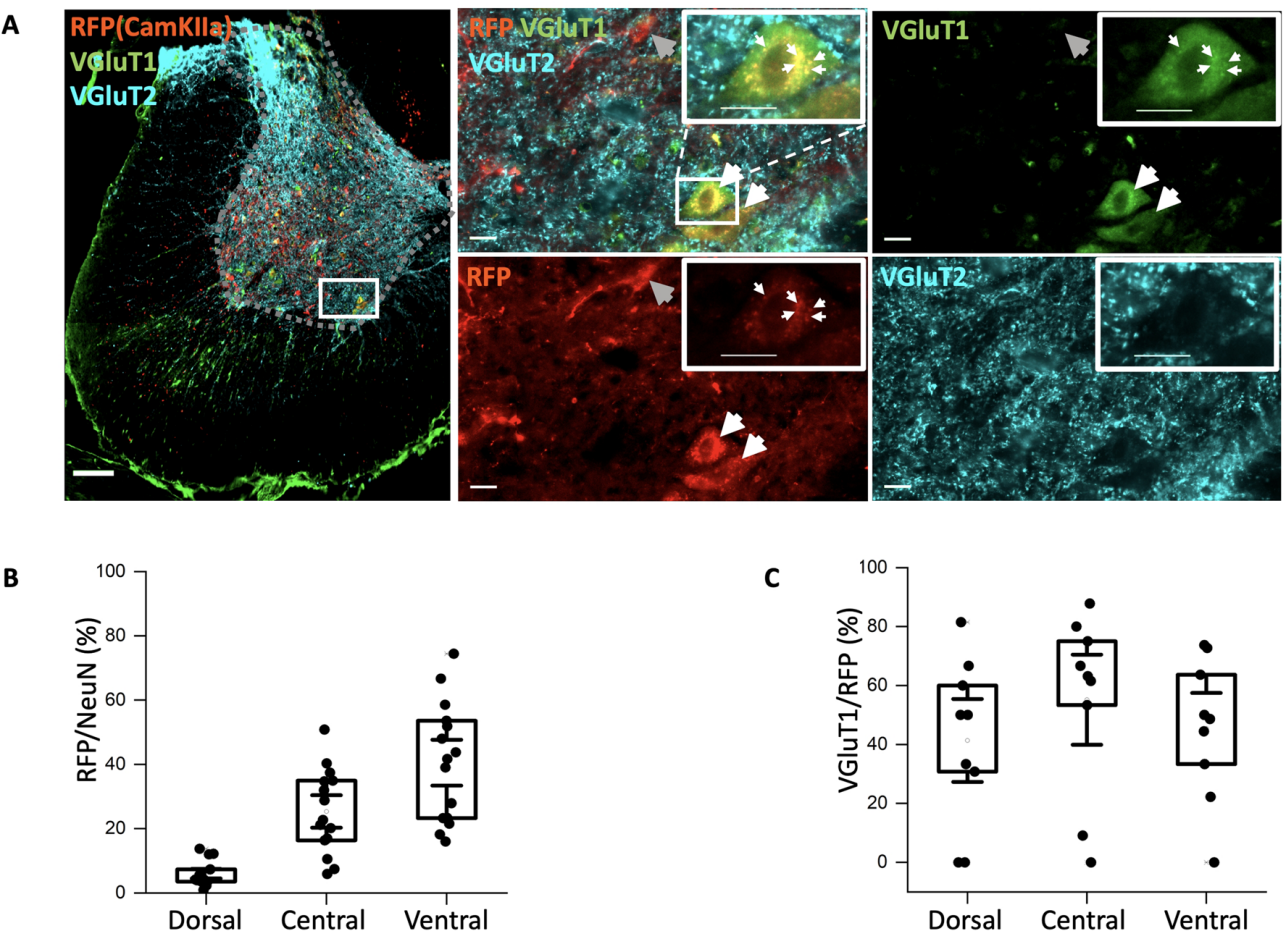
Infection of spinal neurons with AAV9-CamKIIa primarily in the central and ventral regions. (**A**) Overview of infection (red, mScarlet designated here as RFP) in the half spinal section combined with the immunostaining of VGluT1 (green) and 2 (cyan) and zoomed in images of a highlighted region in half section and the immunostaining of VGluT1 and 2 (green and cyan), indicates some overlap with VGluT1, whereas the VGluT2 primarily stains the synaptic terminals. (**B**) Counting neurons (NeuN) that co-expressed with the cells expressing RFP (N = 6 animals, n = 3 sections/animal). (**C**) Box plot of the co-expressed cell count with VGluT1 positive neurons (N = 3 animals, n = 3 sections/animal). Scale bar 200 $\upmu$m in image A (left) and 20 $\upmu$m in the insets.

**Figure 5:**
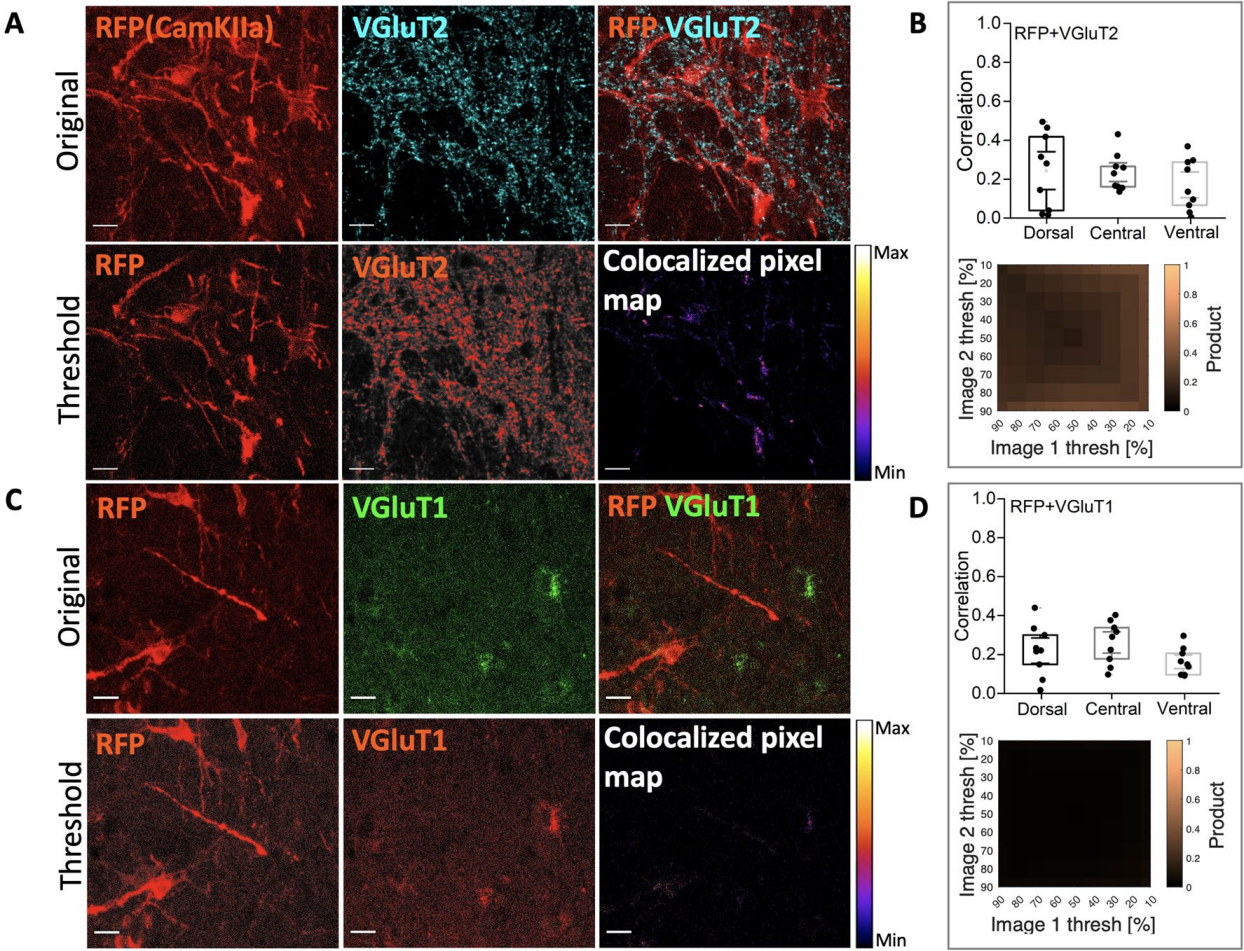
AAV9-CamKIIa has little or no colocalization with VGluT2 and VGluT1 staining. (**A**) Top row: the infected cells with the CamKIIa promoter (AAV9) express mScarlet (RFP) and its overlap with VGluT2 (cyan), and second row: thresholded views of the top row and the heat map of RFP and VGluT2. (**B**) Top right grey box: fluorescent correlation plots of RFP and VGluT2 show very little co-expression. (**C**) First row: original images showing mScarlet (RFP), VGluT1 (green) and their overlap, and bottom row: the threshold views of the images and and the heat map for co-localization. (**D**) Bottom right grey box: the correlation for various levels of threshold appear dark. (N = 3 animals, n = 3–4 sections/animal). Scale bar 20 $\upmu$m.

**Figure 6:**
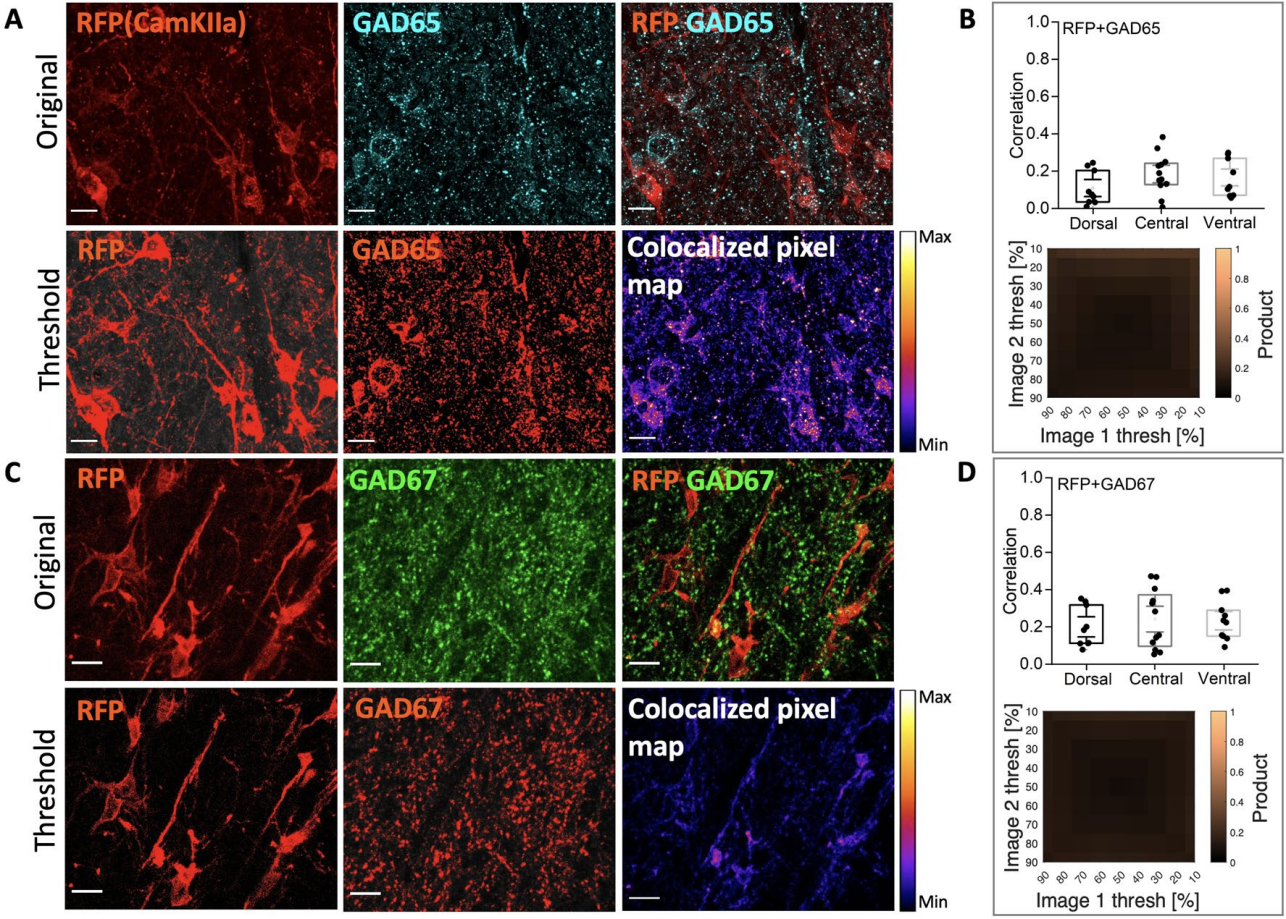
AAV9-CamKIIa-infected cells has poor overlap with GAD65 and GAD67 biomarkers. (**A**) Top row: mScarlet expression (red, RFP) and the immunostaining of GAD65 (cyan) has only little co-expression, and second row: threshold representations of the original images and a heat map co-expression plot of RFP/GAD65. (**B**) Top right grey box: plots show poor correlation in pixel-overlap between RFP and GAD65. (**C**) First row: original images of RFP, GAD67 (green) and their overlap, and bottom row: threshold representations of the original images and the heat map of RFP/GAD67. (**D**) Bottom right grey box: the mosaic of correlation coefficients for various thresholds, indicate poor overlap between RFP and GAD67. (N = 3 rats, n = 3-4 sections/rat). Scale bar 20 $\upmu$m.

### AAV9-CamKIIa targets some excitatory neurons in the spinal cord

Besides investigating the expression of the AAV2-mDlx, we also inspected the expression using the CaMKIIa-promotor with the AAV serotype 9. We used a different fluorescent reporter for the expression of AAV9-CaMKIIa, which is a red fluorescence protein (mScarlet). To determine the fraction of neurons that had expression of mScarlet, we labeled the neurons using NeuN biomarker and counted the fraction that co-expressed in D, C, and V regions (Fig. 4A,B). We identified roughly 5% mScarlet+ neurons in the D, 25% in the central and 40% in the ventral region (Fig. 4B). Next, we tested whether the mScarlet+ neurons had a potential overlap with proteins that are distinctly associated with glutamatergic neurons. First, we tested for overlap with the vesicular glutamate transporters 1 and 2 (VGluT1 and VGluT2) (Fig. 4C,D). However, these transporters are primarily marking the axons and synaptic terminals^21^, which make it difficult to identify the type of cell. Anti-VGluT1 seemed to have occasional staining within soma (top left, Fig. 4C) but the VGluT2 was largely indicated on dendrites outside the soma (bottom right, Fig. 4C). Counting the mScarlet+ cells that receive synaptic contacts we found 50-60% of these receiving inputs from VGluT1 and majority of mScarlet+ neurons receive inputs from VGluT2. VGluT2 is abundantly present in the gray matter, hence resulting in a nebulous staining in the space surrounding the somata (Fig. 4A, bottom right). The colocalization was further quantified by fluorescent correlation as above (Fig. 3). The colocalization using the threshold correlation was low regardless of choice of threshold (Fig. 5). This was, however, expected even if the mScarlet positive cells were VGluT2 positive since there is a medium to low expression of mScarlet in the synaptic terminals.

Another indicator of glutamatergic input to neurons is antibodies for the AMPA receptors, GluA1 and GluA2 (Supplementary Fig.  4). We observed that around 20–30% of mScarlet+ neurons contain GluA1 (Supplementary Fig.  4A–C) at the intracellular sites however it was challenging to trace the co-expression GluA2 with RFP+ neurons as GluA2 was also excessively present (Supplementary Fig.  4B bottom right image) in the spinal cord. These data demonstrate that the given AAV virus with CaMKIIa promoter receives glutamatergic inputs and GluA1/2 expression at various intracellular sites.

To inspect whether some of the AAV9-CamKIIa-driven infection was occurring in inhibitory neurons, the infection was compared with immunostaining of GAD65 and 67. There was some overlap (correlation < 0.2), but not as strong as with the AAV2-mDlx-driven neurons (compare Fig. 3 and 6).

### Comparison between AAV2-mDlx and AAV9-CamKIIa expression

The viral infection with AAV2-mDlx and AAV9-CamKIIa exhibited differences in expressions, which inspire hope that these could be used to differentiate between excitatory and inhibitory spinal interneurons. We compared the ratio of infected GAD65+ cells to cells that were GAD65− by counting the infected cells that colocalized with NeuN in the three spinal regions: Dorsal, Central, and Ventral. To avoid cross-talk between the antibodies that had primary antibodies extracted from the same host organism, we co-stained NeuN and GFP (mDlx, Fig. 2C) in one set of spinal cord sections and GAD65 and GFP in another set of spinal sections (mDlx, Fig. 2D). By counting the number of neurons in these two sets we estimated the percent of expression in GAD65+ neurons using AAV2-mDlx normalized by NeuN+ neurons (Supplemental Fig.  5A) and shown as box plots. Similarly, we calculated percent of GAD65+/NeuN using AAV9-CamKIIa (Supplemental Fig.  5A). These data demonstrate, although there was AAV9-CamKIIa-driven expression in GAD65+ cells the ratio of GAD65+/NeuN was indeed larger when we used AAV2-mDlx compared to the AAV9-CamKIIa in all regions.

### Targeting neurons using AAV9-hSynapsin1

**Figure 7:**
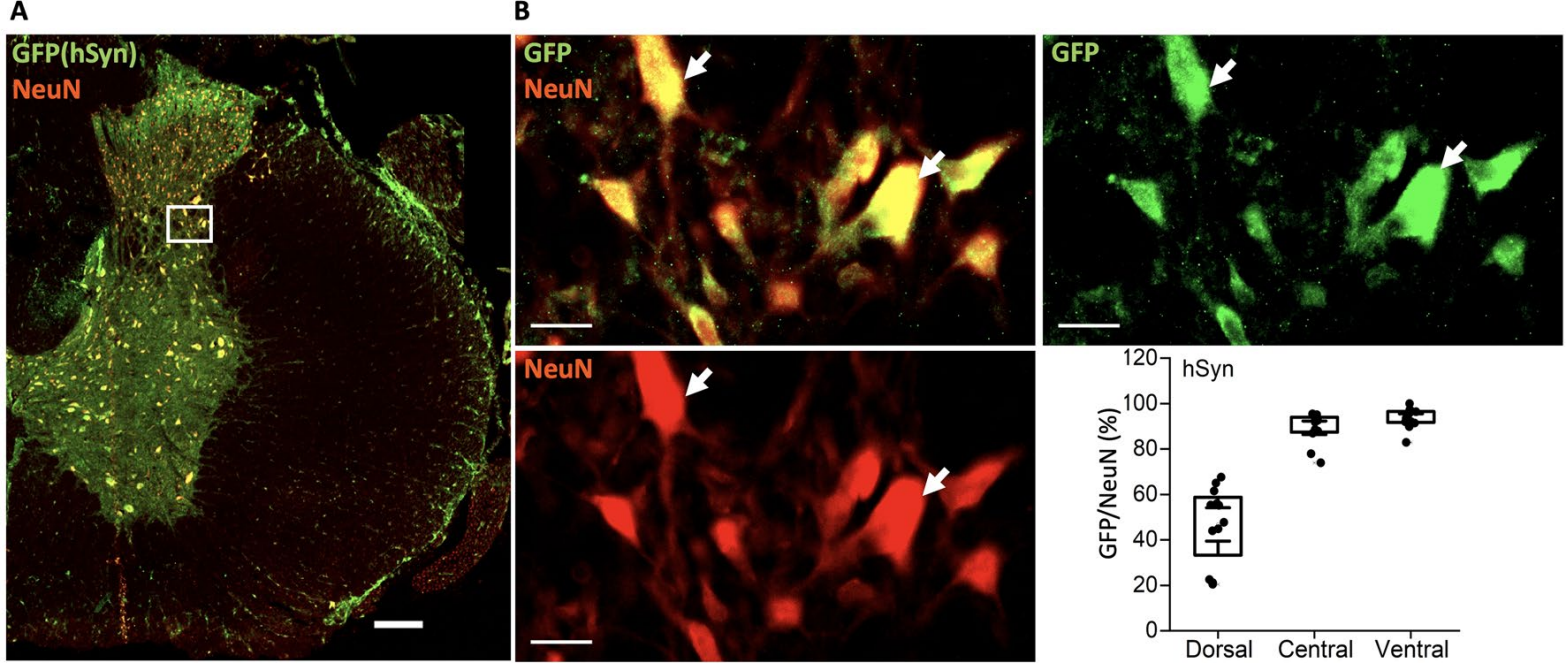
AAV9-hSyn1 targets a large fraction of spinal neurons. (**A**) A spinal section with infection using hSyn1-promoter co-stained with NeuN marker for neurons. (**B**) Highlighted region in (**A**) shows the extensive overlap between the infected cells and the NeuN marker and box plots at the bottom right show 40–60% co-expression of NeuN and GFP in the dorsal region whereas 90% overlap in the central and ventral regions. Scale bar = 200 $\upmu$m in image (**A**) and 20 $\upmu$m in the zoomed-in images (**B**). N = 4 animals, n = 3 sections/animal.

**Figure 8:**
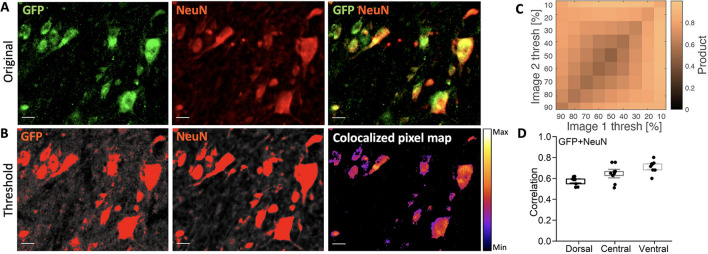
AAV9-hSyn1-driven infection correlation with NeuN biomarker. (**A**) Top row: expression of GFP with hSyn1 promoter has high overlap with the NeuN staining. (**B**) Threshold and co-localization in a heat map. (**C**,**D**) The co-localization is quantified for different thresholds and across the three regions. Scale bar 20 $\upmu$m. N = 3 animals, n = 3 sections/animal.

We also tested the expression using the AAV9-hSyn1 in the spinal cord with expression of GFP and immunolabelling with NeuN. We observed that amongst total NeuN+ neurons nearly 45% of the GFP+ neurons in the dorsal region and 90% in the central and ventral regions were showing co-expression with NeuN (Fig. 7). The extent of the colocalization was further quantified similar to the above using the threshold of the fluorescent signal (Fig. 8) and found similar strong correlation as in Fig. 7. These data indicate most of the spinal cord neurons could be targeted using AAV9-hSyn1.

We further tested the colocalization of cells with AAV9-hSyn1-driven expression with GABAergic antibodies such as GAD65, 67 (Supplemental Fig.  6A) and Pax2 (Supplemental Fig.  6B). Viral expression that was driven by hSyn1 promoter) showed high colocalization with GAD65+ immunolabelling and medium correlation GAD67 and Pax2 immunostaining.

### Few motoneurons have expression

We compared AAV2-mDlx (Supplemental Fig.  7A), AAV9-CamKIIa (Supplemental Fig.  7B) and AAV9-hSyn1 (Supplemental Fig.  7C) expression to investigate their expression in lumbar spinal motoneurons (MN, Supplemental Fig.  7). ChAT-immunolabeling/immunolabelling of neurofilament (SMI32) was used to label MN. We found low expression of all three viruses in motoneurons (Supplemental Fig.  7D).

## Discussion

Spinal interneurons are very diverse and there are many subtypes of GABAergic interneurons involved in e.g. the motor circuitry^13,14,22^. For instance, the V1 inhibitory interneuron is genetically diverse with more than 50 sub-populations^23^ and tools investigating their function are in demand, especially for verification of the translational potential of the experiments done in transgenic model species. Here, we test three enhancers in conjunction with AAVs for specificity in well-defined subtypes of neuron. The plasmids were delivered using adeno-associated virus injected into the lumbar enlargement of rats. We found that the AAV2-mDlx does not induce detectable expression in GABAergic neurons in the superficial layers that are known for high content of both GAD65 and GAD67^24,25^. In the central and ventral layers, where there are less dense labeling there was a considerable co-expression of somata that had strong labeling of GAD65 rather than GAD67 and the reporter protein (GFP). GABAergic neurons in central regions have previously been identified and suggested to involve in sympathetic inhibition^26^. Nevertheless, there was some low correlation found in motoneurons using the AAV2-mDlx. Similarly, low fluorescent correlation was observed in motoneurons using AAV9-CamKIIa. AAV9-hSynapsin1 seems to target most of the spinal neurons still their correlation with motoneurons was low. We also found that the AAV2-mDlx could induce viral expression in some glutamatergic neurons (GluA1-positive). It would be interesting to test other promoters like the *mGAD65*^27^ for specificity in spinal interneurons.

CamKIIa can translocate to excitatory neuronal synapses^28^ as demonstrated in our data where CamKIIa promoter expresses in some GluA1 and VGluT1 neurons (using AAV9) whereas most of the neurons are receiving VGluT2 inputs. To properly identify the infected cells, future studies with in-situ hybridization to detect mRNA for VGluT2^29^ are crucial. CamKIIa can also translocate to inhibitory synapses^28^ and can phosphorylate inhibitory receptors^30–33^ as shown in the fluorescent correlation analysis where, we found some correlation of AAV9-CamKIIa with GABAergic markers such as GAD65 and 67.

One potential issue that has previously been of concern regarding viral infections is the specificity may be dependent on the volume of virus that is present in the extracellular space. However, we do not think this is a major concern, since if there was a strong dependence of specificity on the amount of available virus and the specificity would have decreased with the distance from the injection site and we should have seen less specificity at a few hundreds of microns from the injection site, i.e. more variety in neuronal types, than at the periphery of the sphere of injection which have not been observed in our case. We see that the GAD65 and GAD67 (Fig. 2) did not seem to have more co-localization clustered near the center of injection. When using AAV9-CamKIIa (Fig. 6), there also does not seem to be a difference in the dorsal, central and ventral regions on the pixel map correlations. Similar conclusions can be drawn from Supplemental Figs.  3,  6 and  7. It has previously been reported that excitatory/inhibitory preference of AAV/lentivirus changes by the titer of the viral solution^34^. The expression using systemic administration (AAV PHP.EB) have been demonstrated to depend on the titer and high titer showed significant virus expression in the neuronal cell bodies^35^. Therefore we used high infectious titers in the range of $1 \times 10^{12}$ genome copies/ml throughout the experiments.

The diverse expression in different neuronal subtypes could be due to the tropism of the virus themselves, and this should be considered when using a different serotype. Various AAV serotypes in the central nervous system have shown different ability to infect multiple types of cells, i.e. tropism^5,36–41^. In the present study, we used serotypes AAV2 and AAV9. The AAV9 has often been used in the spinal cord^42,43^ and AAV2.1 has shown substantial expression in the mice prefrontal cortex (Supplemental Fig.  2) and has shown considerable expression in different parts of the brain^40^. Hence, the serotype of the AAV is likely as important as the promotor.

## Materials and methods

### Viral constructs

To explore an AAV viral technique and to target specific neuronal types in lumbar spinal cord of wild-type rats we used three different types of AAV viruses: AAV2.1-mDlx-GCaMP6f-Fishell-2, AAV9-CamKIIa-ChrimsonR-mScarlet-Kv2.1 and AAV9-hSynapsin1-soCoChR-GFP. The AAV2.1-mDlx-GCaMP6f-Fishell-2 was produced by incorporating the plasmid pAAV-mDlx-GCaMP6f-Fishell-2, which is commercially available (Addgene.org)^12^ into a adeno-associated virus of serotype 2. The pAAV-mDlx-GCaMP6f-Fishell-2 was a gift from Gordon Fishell (Addgene plasmid 83899; http://n2t.net/addgene:83899; RRID:Addgene_83899). The pAAV-CamKIIa-ChrimsonR-mScarlet-KV2.1 was a gift from Christopher Harvey (Addgene plasmid 124651; http://n2t.net/addgene:124651; RRID:Addgene_124651), which was incorporated into a AAV of serotype 9. The pAAV-hSynapsin-soCoChR-GFP was a gift from Edward Boyden (Addgene plasmid 107708; http://n2t.net/addgene:107708; RRID:Addgene_107708), which was incorporated into an AAV of serotype 9. In all injections the titer of the virus was $1 \times 10^{13}$ infectious units per mL.

### Laminectomy and virus injection

Rats were anesthetized under isofluorane (1–3% in oxygen) and placed in the stereotax frame on a heating pad with temperature controller. Laminectomy was performed at the thoraco-lumbar (T12–L1) part of the vertebrae^44^, where similar albeit alternative procedure has recently been suggested in mice^45^. The animals received an injection of AAV virus (1500–2000 nl) dorso-ventrally and rostro-caudally at the rate of 15 nl/min using a sharp glass capillary (inner diameter = 0.53 mm and outer diameter = 1.14 mm, Neurostar GmbH, Tubingen, Germany), which was left in place for 3–5 min after the injection to prevent the backflow^46^. Laminectomy site was covered, skin was sutured and the rats were housed in their cages for recovery. Post-operative care was provided via buprenorphine mixed with nutella (sublingual tablets 0.2 mg crushed to powder and mixed with 1 g nutella, Dose = 0.4 mg/kg, every 12 h for 3 days) and carprofen (subcutaneously (s.c.), 5 mg/kg, once a day for 5 days) as analgesic and anti-inflammatory and baytril (s.c., 5 mg/kg, once a day for 10 days) as antibiotic drugs. Animals used in the study showed no signs of distress and were monitored twice a day for 3 consecutive days and later once a day for 7 days.

For further confirmation of AAV2-mDlx expression, fixed brains of C57BL/6N mice with mDlx injection in the prefrontal cortex were provided (as gift) by Dr. David Paul Drucker Woldbye and Amaia de Diego Ajenjo. Brains were sectioned and sections were visualized under the fluorescent microscope.

### Western blot

Four weeks after the viral injection, the rats were deeply anesthetized and euthanized by cervical dislocation. Then the tissue samples were promptly collected on ice and reserved at − 80 $^{\circ }$C until further usage. The samples were homogenized with 1X RIPA lysis buffer (50 mM NaCl, 150 mM Tris-HCl at pH 7.5, 5 mM EDTA, 0.5% sodium deoxycholate, 1% NP-40, 0.1% sodium docecyl-sulfate), stirred (30 min, 4 $^{\circ }$C) and centrifuged at 20,000*g* (20 min, 4 $^{\circ }$C). Protein quantification was performed using the bicinchoninic acid (BCA) method. 30 $\upmu$g protein was loaded per well and the concentration was normalized using loading buffer (2xSDS buffer: 2M DTT, 5xSDS, MilliQ) to reach 25 $\upmu$l total volume. Samples were vortexed and boiled for 6 min. Samples were loaded and electrophoresed in a 12% SDS-polyacrylamide gel (Bio-Rad Laboratories, CA, USA) and transferred to the membrane (PVDF, Immobilon-P, Millipore, Billerica, MA, USA). The membranes were blocked in 5% milk in Tris-buffered saline Tween-20 (TBST), 1 h at room temperature. The following primary antibodies were applied for overnight at 4 $^{\circ }$C: rabbit anti-beta actin (1:100,000, A3854 Sigma) and chicken anti-GFP (1:5000, AB13970, Abcam). Secondary antibodies were applied the next day: HRP donkey conjugated-anti-rabbit (1:10,000, 31430 Thermo Scientific) and IgY (H+L) goat anti-chicken cross-adsorbed antibody, (1:500, Alexa Fluor Plus 488 Thermo Scientific). Finally, membranes were washed 5 times in the washing buffer with TBST every 5 min and developed. The target proteins were detected by chemiluminescence (ECL).

### Immunohistochemistry

A month after virus injection, rats were deeply anesthetized using pentobarbital administered intraperitoneally (i.p) and transcardial perfusion was performed using PBS followed by 4% paraformaldehyde (PFA). The fixed spinal cords were extracted and kept in PFA for 4 h followed by 30% (w/v) sucrose for cryoprotection and later use^47^. 20 $\upmu$m transverse lumbar spinal slices were cut using cryostat, collected on Superfrost plus glass slides (Thermo Fisher Scientific GmbH, Germany) and stored at − 20 $^{\circ }$C. The whole procedure was performed under moist conditions in dark and was obtained from prior reports^48,49^. The spinal slices were washed, incubated with blocking solution (5% fetal bovine serum, 5% bovine serum albumin, 1% PBS, 0.3% Triton X-100) at room temperature for 1–2 h and primary antibodies were applied for overnight (4 $^{\circ }$C). The following antibodies were used such as NeuN (rabbit monoclonal; 1:500 dilution; abcam AB177487), anti-GFP (chicken polyclonal, 1:1000 dilution; abcam AB13970), GAD65 (rabbit polyclonal; 1:500 dilution; Sigma G5038), GAD67 (mouse monoclonal; 1:1000 dilution; abcam AB26116), Pax2 (rabbit polyclonal; 1:500; Invitrogen UD283859), SMI32 (mouse monoclonal; 1:1000 dilution; EMD Millipore; 3256992), ChAT (goat polyclonal; 1:500; EMD Millipore AB144P), VGluT1 (mouse, 1:1000 dilution, synaptic Systems, GmbH, 407 Goettingen, Germany, 135011), VGluT2 (rabbit monoclonal; 1:500 dilution; abcam ab216463), GluA1 clone C3T (rabbit monoclonal, 1:500; EMD Millipore 04-855), GluA2 (mouse monoclonal; 1:200; EMD Millipore MAB397). Subsequent day, the slices were washed and incubated with secondary antibodies (1:500 dilution), donkey anti- rabbit Alexa Fluor 594 (Invitrogen R37119), donkey anti-chicken Alexa Fluor 488 (Jackson ImmunoResearch 703-546-155), donkey anti-mouse Alexa Fluor 647 (Invitrogen, A31571), donkey anti goat (abcam AB150132) and DAPI (1:1000 dilution applied for 2 h; Sigma-Aldrich) at room temperature for 1–2 h. Mounting was performed using DAKO mounting medium and the slices were visualized using Axio scan.Z1 and confocal microscopes using 20× magnification. Captured images were finally processed using Zen Lite 3.1 and ImageJ softwares.

### Quantification and statistics

Quantification to verify the AAV viral co-expression with neuronal biomarkers was performed by manually counting the neurons in the D, C and V spinal cord followed by computing the percentage of co-expression. The co-expression was further computed by using the colocalization threshold and Ezcolocolization plugins in ImageJ^20^. Ezcolocalization can be used to perform multiple types of image analysis such as: (1) selecting the cells or any tissue structure based on the fluorescence intensity, (2) measure correlation coefficient of two images by using fluorescence intensity thresholds, (3) create heat maps, metric matrix and scatter plots. Pearson correlation coefficient was computed using just another colocalization plugin (JACoP)^50–52^. Graphs were made using MATLAB and commercially available software (Origin2020, Origin Lab) and the plotted values are either shown as percent change of a new value with respect to the original value or as fluorescence correlation.

### Animals and ethical statement

The study is reported in accordance with ARRIVE guidelines^53^. All experiments were approved by the Danish veterinary and food administration (animal research permission number 2019-15-0201-00018) and according to guidelines of the Council of the European Union (86/609/EEC).

Wild-type adult Wistar rats obtained from Charles River Laboratories were used for the experiments. Animals were housed in pairs at the animal facility and maintained in 12 light/12 dark cycle. Male rats were used however sex was not a crucial element of the study. Animals were habituated for at least 10 days before starting the experiment.

## Supplementary Information

Supplementary Information.
