# Supplementary material for: Viral strategies for targeting spinal neuronal subtypes in adult wild-type rodents
Source: Sci Rep. 2022 May 23;12:8627. doi: 10.1038/s41598-022-12535-4 (PMC9126985; doi:10.1038/s41598-022-12535-4)
Supplement: Supplementary file 1 — Supplementary Information. [file 41598_2022_12535_MOESM1_ESM.pdf]

Supplementary Information

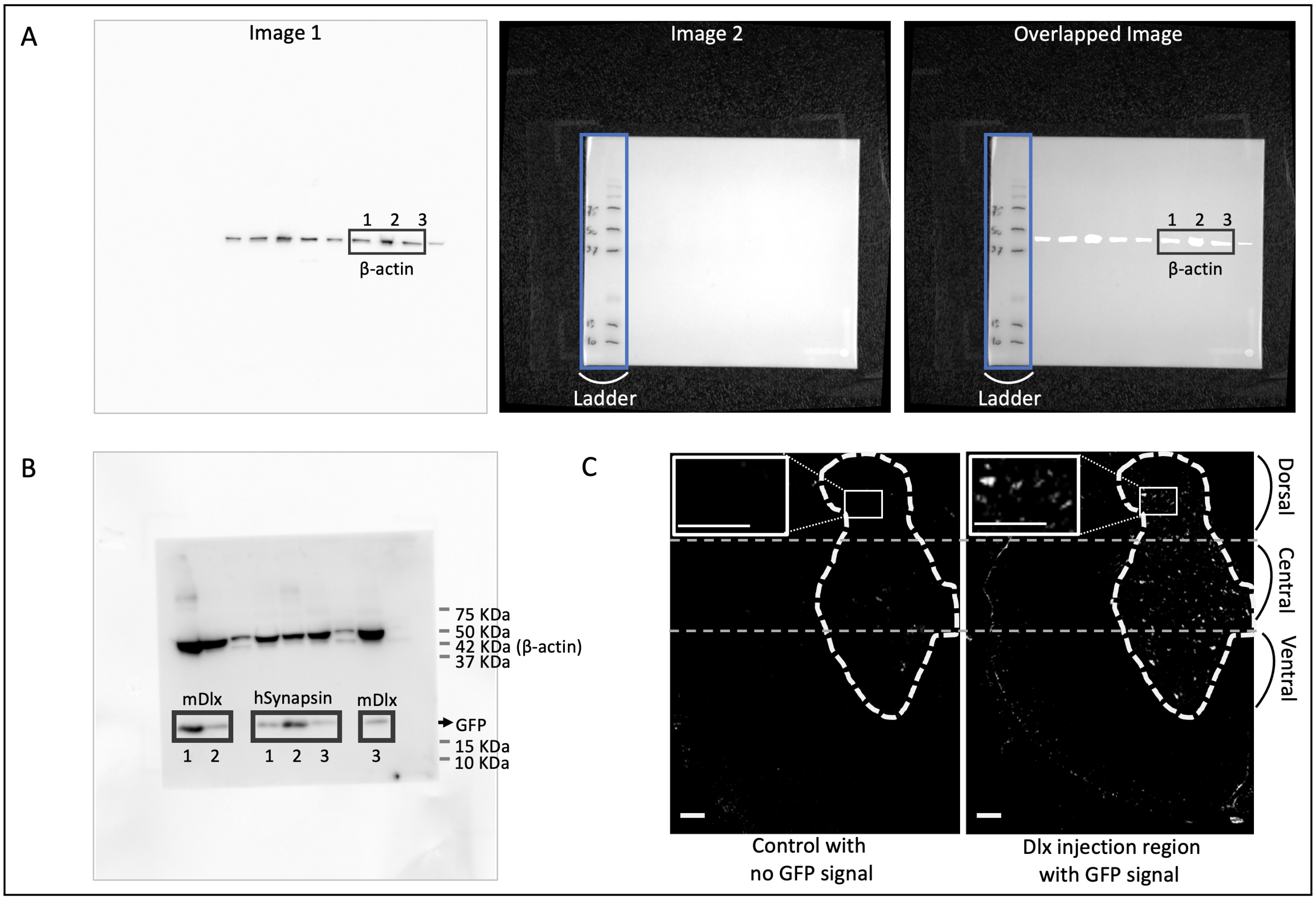

Supplementary Figure 1. Western blots (A and B) shown for promoters mDlx and hSynapsin1 and an alternate way (C) of confirmation of AAV virus in the spinal cord sections. **(A)** Full blot of beta-actin (image 1), a ladder was highlighted in blue (image 2) and an overlap of beta-actin (image 1) was shown with a ladder image (image 2). The edges of full length western blot beta-actin image (image 1) were not visible and therefore to make the edges visible, a ladder (image 2) made for this beta-actin blot was overlapped with image 1. **(B)** Full western blots of AAV2.1-mDlx and AAV9-hSynapsin viruses were shown where the replicates were represented by the numbers - 1, 2 and 3 (B). Note that black boxes in the images represent the cropped images shown in the main text of Figure 1. Bands at 50 KDa (B) were not taken into consideration here. **(C)** Alternatively AAV virus injection and expression was confirmed by sectioning the spinal cords and directly observing the sections under fluorescence microscope as shown here in the example images where AAV2.1-mDlx virus expression was visualized in comparison to control spinal cord with no virus expression. The image on the right also shows spread of the AAV2.1-mDlx virus injection. Scale bars: 200 μm and 20 μm in the zoom-in images (C).

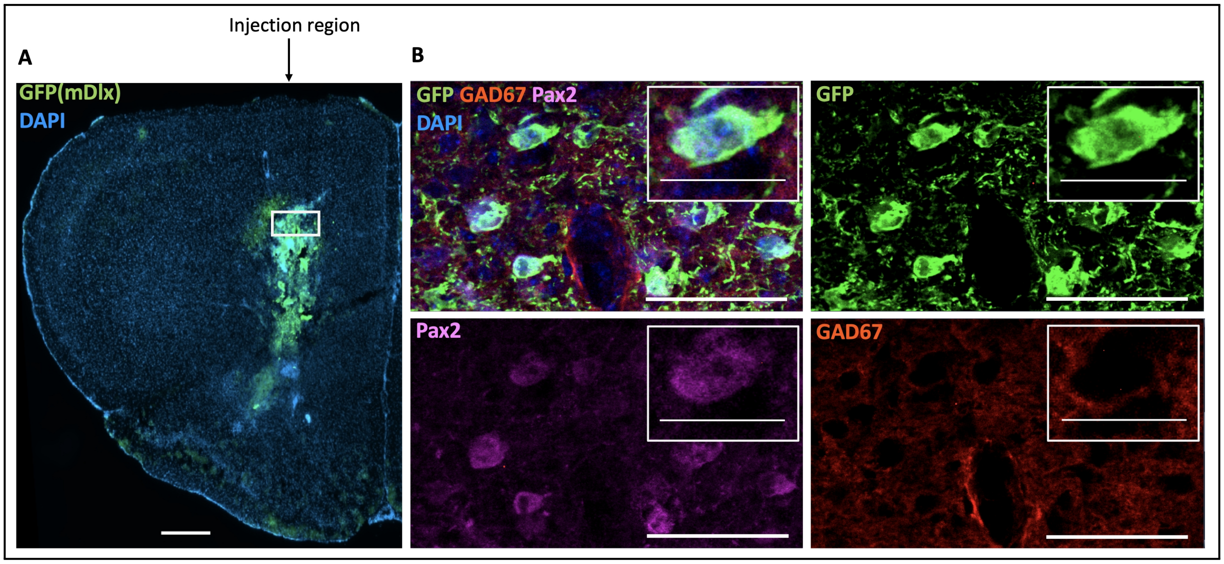

Supplementary Figure 2. **AAV2-mDlx expression in the mouse brain**. **(A)** Coronal section of mouse prefrontal cortex (Scale bar: 500 μm) where the green labelling (GFP) indicates injection spread and expression of AAV2 virus with mDlx promoter. **(B)** Highlighted region in (A) of viral expressed cells labelled by GFP showed colocalization with Pax2 while GAD67 was only weakly colocalized (red). Scale bars: 50 μm in image B and 20 μm in the zoomed-in images which represent single cell (B). N = 3 mice, n = 3-4 sections/mouse.

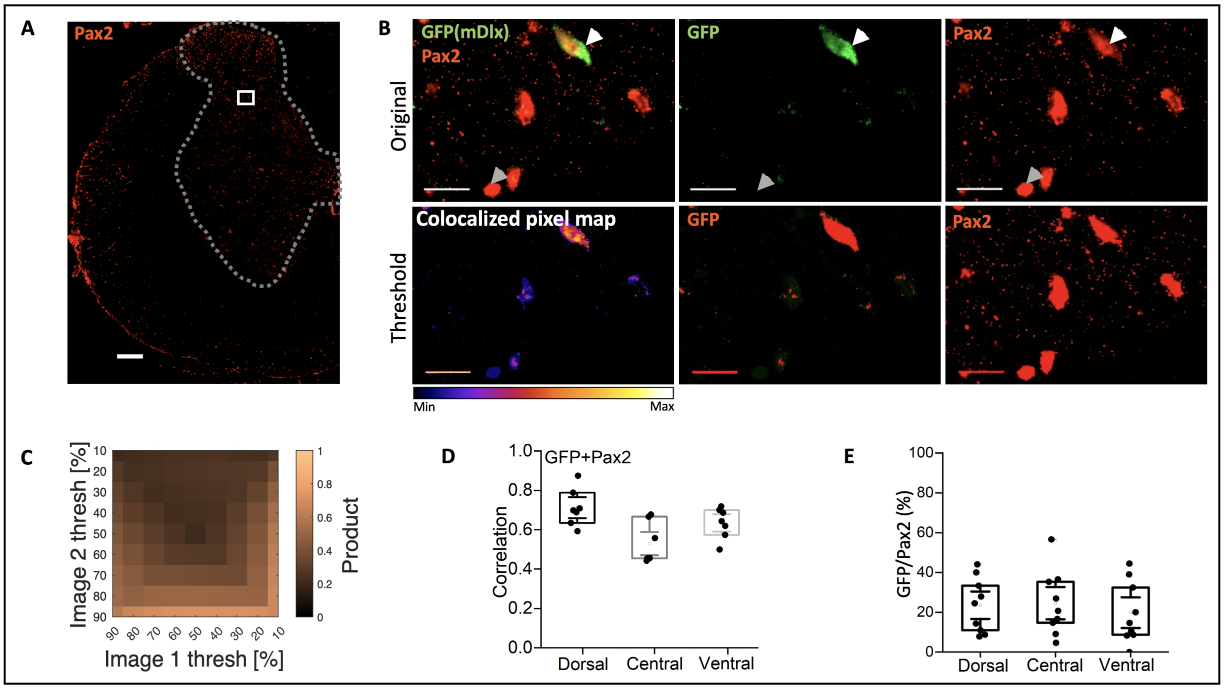

Supplementary Figure 3. **AAV2-mDlx has some co-expression with Pax2**. **(A)** Transverse section of a half lumbar spinal cord indicating an inset region. **(B)** Combined fluorescent images of the GFP-positive neurons (green), where the virus infected the cells and the Pax2 positive immunostaining (red). **(C)** A threshold plot was created by calculating fluorescent correlation from the images. **(D)** The correlation of the GFP and Pax2 fluorescent signals in the dorsal, central and ventral regions. **(E)** Fraction of Pax2+ cells with viral expression (GFP) out of all GFP-expressing cells in percent. Scale bars: 200 μm in image A and 20 μm in the zoom-in images (B). N = 3 rats, n = 3 sections/rat.

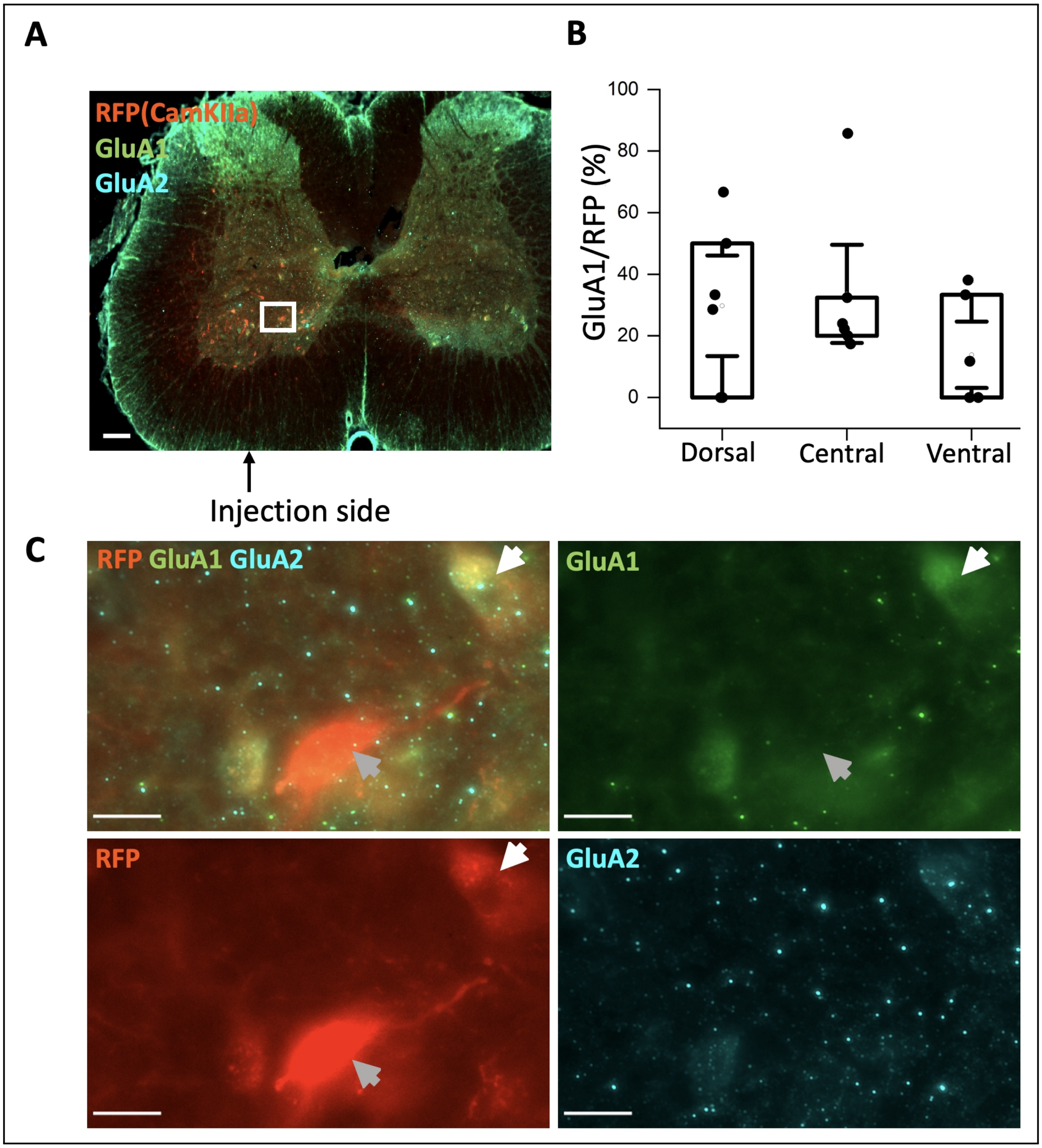

Supplementary Figure 4. The neuronal expression of mScarlet via AAV9-CamKIIa has some co-expression with GluA1 neurons. (**A**) Overview showing the virus injection region and infected cells in red (designated as RFP). (**B**) Fraction of cells positively identified with GluA1 (glutamatergic neuron) out of all cells expressing the virus in percent in the three regions (N = 3 rats, n = 3 sections/rat). (**C**) Highlighted region in (A) with GluA1 (green) and 2 (cyan). Scale bars: 200 μm in image A and 20 μm in the zoom-in images.

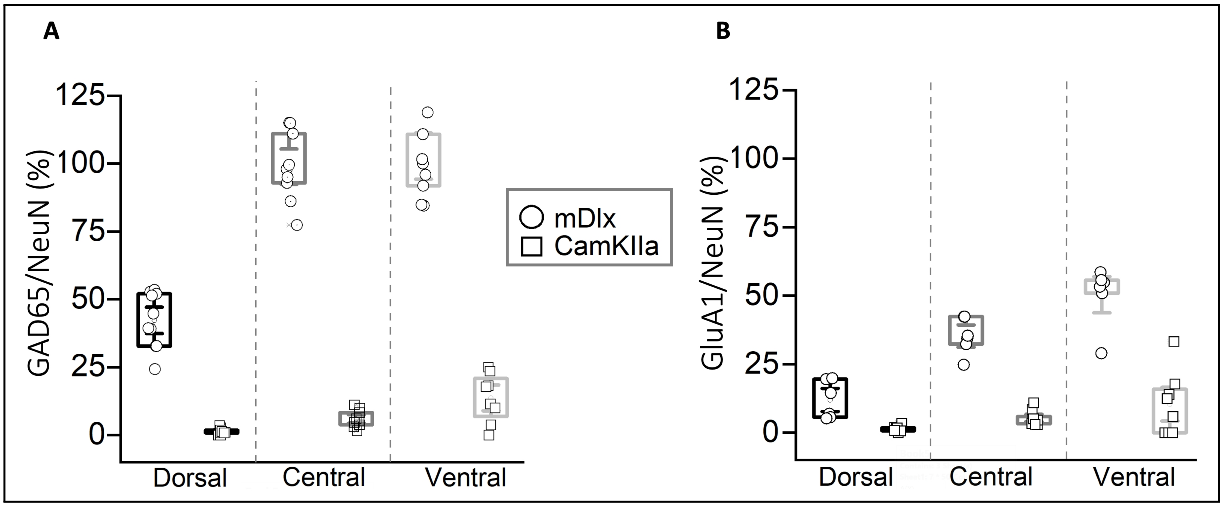

Supplementary Figure 5. Ratio of expression in GAD65+ and GluA1+ cells to the total number of viral expressing cells as counted by colocalization with NeuN using mDlx promoter versus CamKIIa promoter. **(A)** Ratio of GAD65+ cells using either of the promotors (mDlx or CamKIIa) to the total number of viral expressing cells, which is assessed by counting the number of NeuN cells that was infected. Due to technical limitations, i.e. the number of fluorescent probes in same slice, the total number of infected NeuN cells were counted in a consecutive section, which may have slightly different number of neurons, hence the fraction could be larger than 100%. N = 3 rats, n = 3-4 sections/rat (mDlx) and N = 2 rats, n = 3 section/rat (CamKIIa) in lumbar spinal cord. **(B)** The ration of GluA1+ cells to total number of viral expressing cells estimated in the same manner as in (A) for either promotors (mDlx and CamKIIa) N = 3 rats, n = 3 sections/rat.

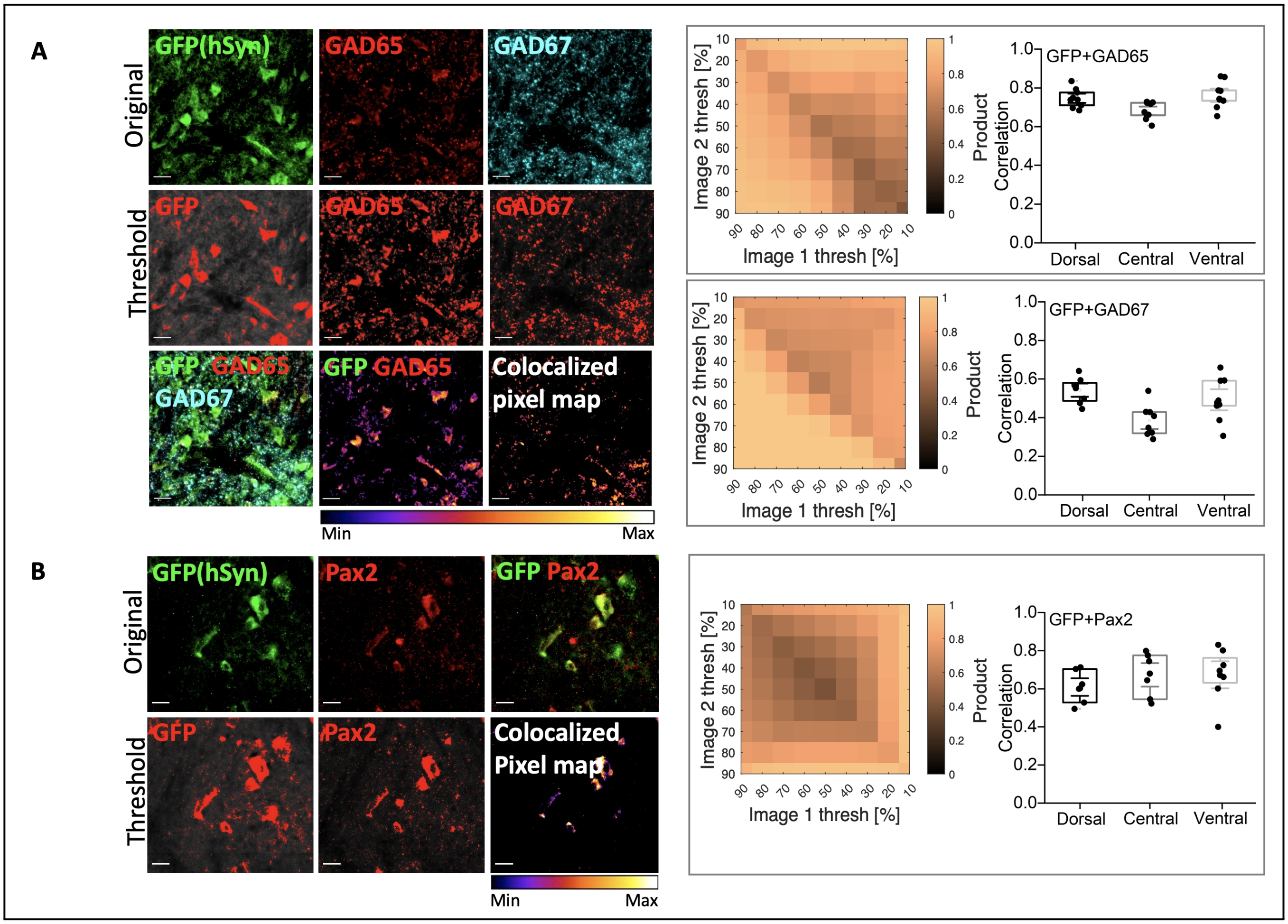

Supplementary Figure 6. **Correlation of hSynapsin1 (designated as GFP) with GAD65, GAD67 and Pax2. (A-B)** Using original images, threshold masks and therefore fluorescent correlation values were computed where the top plot shows correlation of 0.8 between GFP/GAD65 (A), middle plot shows 0.4-0.6 correlation between GAD67/GFP (A) and the bottom plot shows 0.6-0.7 correlation between GFP/Pax2 (B). Co-localized pixel maps show the the ratio of pixels co-localized between GFP and a biomarker such as GAD65 (A), GAD67 (A) and Pax2 (B). Scale bars: 20 μm. N = 3 rats, n = 3 sections/rat.

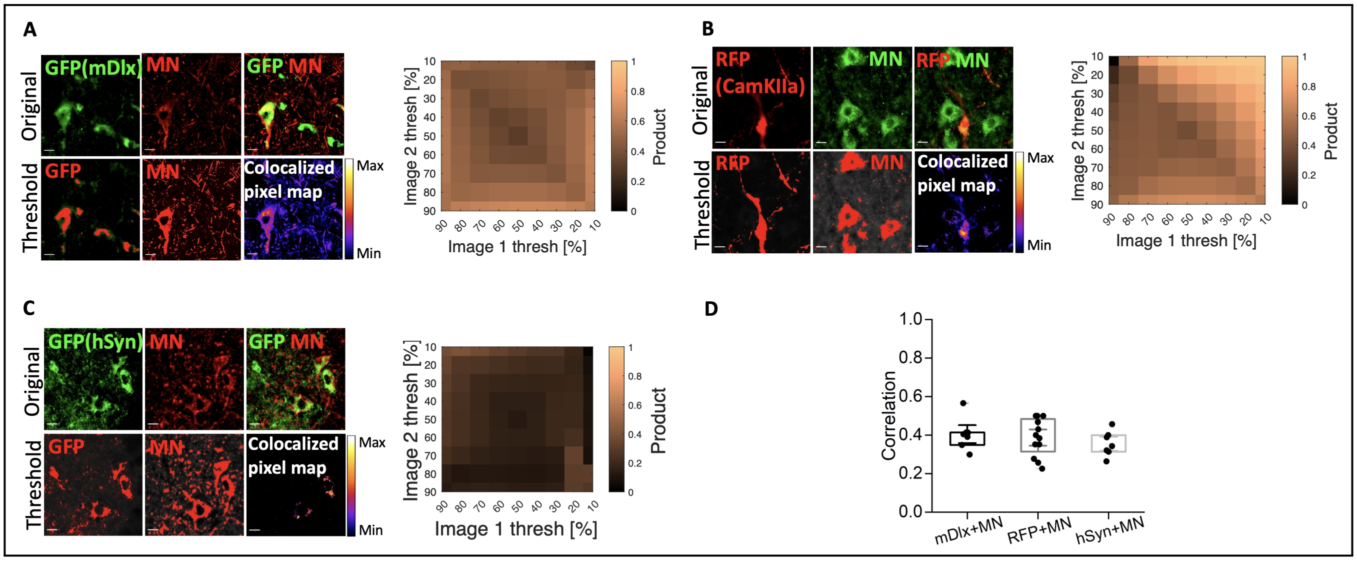

Supplementary Figure 7. Fluorescent correlation of the three viruses with putative motor neurons (by ChAT and neurofilaments SMI32-labeling). (A-D) Threshold values were computed for mDlx (GFP)/MN (A; N = 2 rats, n = 3-4 sections/rat), CamKIIa (RFP)/MN (B; N = 4, n = 3-4) and hSynapsin1 (GFP)/MN (C; N = 3 rats, n = 3 sections/rat) and were shown in their corresponding threshold plots (A-C) and in the form of box plot (D) where mDlx+MN, RFP+MN and hSyn1+MN show correlation of (approximately) 0.4. Co-localized pixel maps (A-C) show the the ratio of pixels co-localized between the virus and the biomarker. Scale bars: 20 μm.
